# Adult Stem Cells and Induced Pluripotent Stem Cells for Stroke Treatment

**DOI:** 10.3389/fneur.2019.00908

**Published:** 2019-08-28

**Authors:** Héctor Fernández-Susavila, Ana Bugallo-Casal, José Castillo, Francisco Campos

**Affiliations:** Clinical Neuroscience Research Laboratory, Health Research Institute of Santiago de Compostela (IDIS), Santiago de Compostela, Spain

**Keywords:** adult stem cell, cell therapy, clinical trial, induced pluripotent stem cell, stroke

## Abstract

Stroke is the main cause of disability and death in the world within neurological diseases. Despite such a huge impact, enzymatic, and mechanical recanalization are the only treatments available so far for ischemic stroke, but only <20% of patients can benefit from them. The use of stem cells as a possible cell therapy in stroke has been tested for years. The results obtained from these studies, although conflicting or controversial in some aspects, are promising. In the last few years, the recent development of the induced pluripotent stem cells has opened new possibilities to find new cell therapies against stroke. In this review, we will provide an overview of the state of the art of cell therapy in stroke. We will describe the current situation of the most employed stem cells and the use of induced pluripotent stem cells in stroke pathology. We will also present a summary of the different clinical trials that are being carried out or that already have results on the use of stem cells as a potential therapeutic intervention for stroke.

## Introduction

From the moment that the capacity of differentiation and self-renewal of stem cells became known, their use as cell therapy for a wide range of diseases has been considered. The international community has focused on this idea, starting a revolution in the study of stem cells (1). This revolution led to several important discoveries that, step by step, paved the way to convert cell therapy into reality. But the greatest discovery was made in 2006 when Yamanaka and Takahashi were able, for the first time, to generate induced pluripotent stem cells (iPSCs) from adult somatic cells by inducing the artificial expression of four transcriptional factors: OCT4, SOX2, c-MYC, and KLF4 (2). This new approach provided a considerable resource of human pluripotent stem cells that could be propagated during long-term culture and yet be differentiated to a variety of lineages representatives of the three embryonic germ layers, solving the ethical limitations caused by the use of human embryonic stem cells.

**Figure 1:**
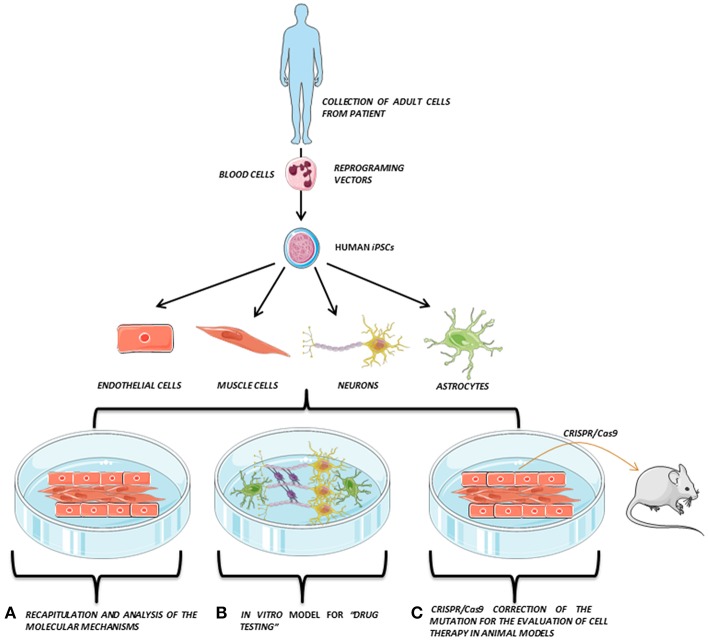
iPSCs modeling scheme. Adult somatic cells (e.g., blood cells) are collected from the patient, reprogrammed and derived to the affected cell types (e.g., endothelial cells, muscle cells, neurons, or astrocytes), which are co-cultured *in vitro*, opening the possibility to perform several studies directly on the patient's own cells. Adapted from Servier Medical Art by Servier is licensed under a Creative Commons Attribution 3.0 Unported License (https://smart.servier.com/).

In addition, the generation of human iPSCs from different somatic cells of patients and the subsequent differentiation to the affected cell lineage has allowed the recapitulation of features of genetic pathologies through *in vitro* disease modeling and the discovery of new treatments directly tested on these human cells. Recently, the combination of iPSCs with the advances in genome editing techniques, such as the clustered regularly interspaced short palindromic repeat (CRISPR) system, has also provided a promising way to repair putative causative alleles in patient lines into a healthy cell line for future autologous cell therapy (3, 4) (Figure 1).

The development of human iPSCs has also opened a new opportunity for those neurological diseases where the affected neuronal type is well-known or the genetic cause of the pathology is well-described such as (i) Alzheimer's (5, 6), (ii) Parkinson's (7, 8), (iii) amyotrophic lateral sclerosis (9), or (iv) Huntington disease (10). In these pathologies, iPSCs have been used to generate neuronal cell lines to recapitulate and study the mechanics of the pathology in *in vitro* models or to evaluate their neurorecovery capability.

**Figure 2:**
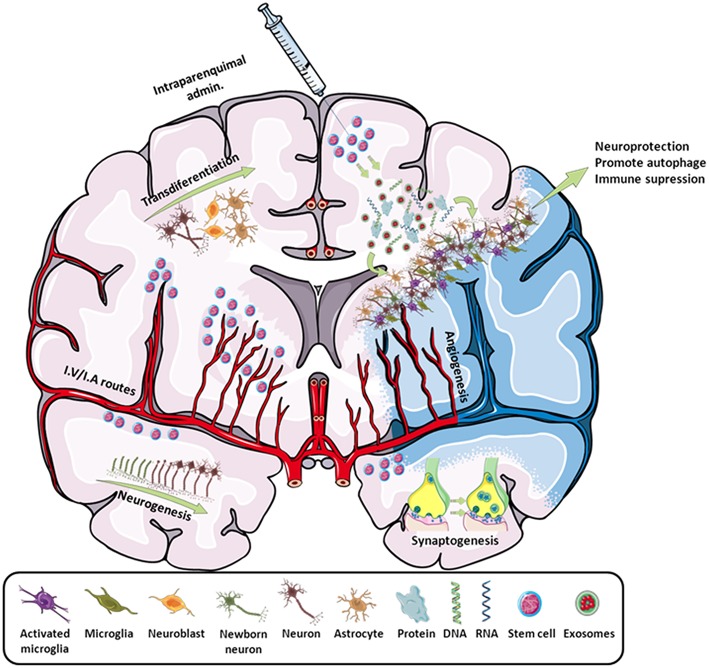
Scheme of all the main effects promoted by stem cells in stroke. By intraparenchymal injection or i.v./i.a. routes, stem cells induce neurogenesis, transdifferentiation, angiogenesis, synaptogenesis, and immune modulation by attracting or releasing trophic substances to the infarcted area. Adapted from Servier Medical Art by Servier is licensed under a Creative Commons Attribution 3.0 Unported License (https://smart.servier.com/).

In the field of stroke, like other stem cells, iPSCs have been used as a neuroprotective cell therapy (mainly based on their immunomodulatory capacity) or as a neuroreparative therapy (by inducing neurogenesis, angiogenesis, synaptogenesis, modulation of the immune response, or transdifferentiation) (Figure 2). Besides its neuroprotective or neuroreparative application, the use of iPSCs for stroke modeling has been poorly exploited mainly because this is a neurological pathology with multiple affected cells types and reduced genetic component, compared to other neurological diseases such as Alzheimer's or Parkinson's. However, the use of iPSCs has been recently explored to model neurovascular pathologies associated with risk of stroke (11, 12), opening a promising approach in the study of these neurovascular diseases.

In this review, we offer a general overview of the use of adult stem cells and iPSCs in stroke, addressing the main problems and the main clinical trials that already present results.

## Adult Stem Cell Therapy in Stroke

Stroke, resulting from the interruption of blood supply to the brain, is the leading cause of disability and death in the world within neurological diseases despite a decrease in its mortality rate (13). Pharmacological or mechanical reperfusion therapies are the most effective treatments during the acute phase of ischemic stroke and it is associated with good outcome in 50–70% of cases. However, these treatments are only applicable to <20% of patients because of the short therapeutic window and side effects (14).

Stem-cell-based therapies have emerged as a promising tool for the treatment of both acute and delayed phases of stroke owing to their multipotentiality, ability to release growth factors, and immunomodulatory capacities. Thus, this transdifferentiation is able to produce cells with a neural lineage; induce neurogenesis, angiogenesis, and synaptogenesis; and activate endogenous restorative processes through the production of cytokines and trophic factors. Moreover, the regulation of cerebral blood flow (CBF), the blood–brain barrier (BBB), and other neuroprotective mechanisms, such as the reduction of apoptosis, inflammation, and demyelination or the increase of astrocyte survival, have also been described as beneficial after stroke (15).

While the technology of the iPSCs is quite new and deeper studies are being carried out to know its real translationality, studies with adult stem cells have been performed for much longer, and there is more information about their use in cell therapy for stroke. Furthermore, there are already clinical trials going on and even closed with adult stem cells. Focusing on stroke, the most frequently used stem cells are the mesenchymal stem cells (MSCs), due to their great trophic capabilities, and the neural stem cells (NSCs), because of their neurorecovery activity (15).

## Methodology in Stem Cell Administration for Stroke

Despite the special attention on stem cells as a promising therapeutic candidate for stroke, parameters such as administration route or cell dosage are still under discussion.

There are relatively few studies that have compared the different possible routes of administration of stem cells. The first studies that used stem cells for cerebrovascular diseases were looking for a neuronal replacement, so they chose an intraparenchymal injection as the most direct route for cell engraftment. These studies showed that the stem cells not only survived but they migrated to the affected zone (16, 17). However, this choice is not the most suitable due to the need of opening a cranial window and also because it damages the brain parenchyma, which is not convenient for stroke patients.

The main alternatives to this route of administration are the vascular routes, either intra-arterial (i.a.) or intravenous (i.v.), which are currently the most used for cell delivery. Intravenous injections are minimally invasive, but cell tracking studies following this route have shown that most administered cells remain trapped mainly in the lungs (18, 19), liver (20), and spleen (21), indicating that a reduced number of cells reach the brain. On the other hand, i.a. administration is a promising strategy to direct the majority of injected cells to the brain, but this is a risky administration route and the fate of injected cells following this route remains unknown due to high variation in the reported results (22).

Whether one route is more efficient than the other is not clear and depends on the cell type used. Thus, in some studies, it was found that the injection of neural progenitor cells (NPCs) by i.a. through the carotid artery presented a higher migration rate in brain and a wider distribution pattern than i.v. administration. Nevertheless, the mortality rate for this i.a. delivery was significantly higher (41%) than in i.v. injection (8%) (23). However, in other studies with bone marrow stem cells (BMSCs) and bone marrow mononuclear cells, there was no greater mortality or greater recovery of infarct volume of one route with respect to the other (24, 25).

The size of stem cells is also a critical parameter when passing through the lungs and should be taken into consideration to decide the best route of administration. As an example, when using MSCs, the majority of them get trapped in the lungs, while the NSCs have a pass-through rate twofold higher (18).

Aimed to clarify the discrepancies about the best route for cell administration in stroke, we have recently reported an experimental study to investigate whether MSCs were able to reach the brain following i.a. or i.v. administration after transient cerebral ischemia in rats and to evaluate the therapeutic effects of both routes (22). Based on our findings, we could conclude that MSCs were found in the brain following i.a. but not i.v. administration in ischemic rats. However, the i.a. route increased the risk of cerebral lesions (microstrokes) and did not improve functional recovery, while the i.v. delivery produced functional recovery and was safe but MCSs did not reach the brain tissue. This fact implies that treatment benefits are not attributable to brain MCS engrafting after stroke (22).

Cell dose is another issue to take into account for both i.a. and i.v. administration that has not been very well-elucidated yet. In line with other studies, we have estimated that doses higher than 1 × 10^5^-0.25 × 10^6^ cells/mL administered i.a. as bolus infusion increase significantly the risk of arterial occlusion (22, 26), while other studies have estimated that doses to 3 × 10^7^ cells are safe (24, 25).

## Stem Cell Fate and Engraftment

It has been widely accepted that there is not engraftment of MSCs in the brain after i.v. administration and that the repair and recovery effects observed in some ischemic animals models are mediated by trophic factors secreted by MSCs (22, 24, 27, 28). However, other studies mainly focused on the intraparenchymal cell injection in brain have found successful engraftment results of cells in the ischemic tissue. The survival and the engraftment rate of the stem cells in brain are critical parameters that determine the successful rate of the repairing and healing effects after cerebral ischemia (29, 30).

Despite the low level of cell brain engraftment, it has been shown that intraparenchymal cells promote neovascularization and functional recovery (31). One of the most relevant studies about this issue was performed by Kokaia's team who carried out a 4-month follow-up of iPSC-derived NSCs transplanted into brain striatum and cortex of rats and mice subjected to stroke. They found that that engrafted cells survive in the brain for up to 4 months without forming tumors, observing a sensorimotor recovery even 1 week after cell administration. In this study, most of the cells had differentiated to neurons able to form axonal prolongations. In addition, the proliferative capacity of these engrafted cells diminished from 40% at 2 weeks to 8 and 0.5% at 2 and 4 months respectively, probably due to this NSC differentiation to neurons (29). The same findings have also been observed when BMSCs were intraparenchymally administered, observing an engraftment of the cells at the ischemic penumbra and at the subventricular zone (32).

Nowadays, cell targeting with contrast agents for the *in vivo* tracking by magnetic resonance imaging (MRI) or positron emission tomography (PET) are being established as powerful technology tools to determine fate and survival after cell treatment, after i.v., i.a., and intraparenchymal cell injection (33).

Indeed, studies using MRI have allowed follow-up of cells for up to 1 year after transplantation (34). One of the most important aspects of the *in vivo* cell tracking is that the cell tracers cannot interfere with the properties and cell viability. In this regard, contrast agents such as gadolinium, superparamagnetic iron oxide agents, or fluorine 19 (35, 36) have been widely used for MRI cell tracking as they do not interfere with cell viability or the migration capacity (33, 37).

Gadolinium (Gd^3+^) has been used to label and track different types of stem cells, such as hematopoietic progenitor cells, monocytic cells, endothelial progenitor cells, and MSCs in small cell transplantation studies. Because they are not nanoparticles, the cellular uptake of Gd^3+^ chelates occurs by pinocytosis (a non-specific form of endocytosis in which small particles present in the extracellular fluid are internalized into cells) or *via* electroporation. However, overall, the low sensitivity of these contrast agents and low uptake by cells are the main limitations to cell labeling with Gd^3+^ (37).

Superparamagnetic iron oxide agents, also known as SPIO nanoparticles, have a much stronger MR sensitivity compared to other paramagnetic agents. Thousands to millions of SPIOs can be internalized in the cells, which makes them generate an MRI signal that is strong enough to visualize a small number of transplanted cells under *in vivo* conditions. The use of SPIOs is mainly restricted to assessing the acute retention of labeled cells and their short-term distribution in the body as these nanoparticles may occasionally diffuse to other tissues or can be scavenged by macrophages, which can then generate a false signal on MRI (37).

Fluorine 19 as a contrast agent for MRI has recently gained more attraction because of several advantages. Fluorine 19 provides a more accurate, unambiguous detection of labeled cells (given the lack of background signal). Moreover, the relationship between the concentration of fluorine 19 and signal intensity is directly proportional and linear over a wide range of concentrations, and the signal can be quantified directly from the acquired images. In addition, the lack of a detectable background in biological tissues leads to higher visibility of the target cells, much like “hot spots” emerging from an empty background (37, 38).

The quantity threshold needed to detect labeled cells is clearly different for SPIOs and fluorine 19. A comparative analysis, using different types of iron-oxide-based contrast agents for MRI labeling of embryonic stem cells, NPCs, and dendritic cells, showed that there is a strong dependence of the iron oxide uptake and label stability on the type of iron oxide particles and cell lines used. Although sufficient uptake was achieved to allow for single-cell detection, in this study, it was estimated that the amount of iron necessary for single-cell imaging was 1–3 pg/cell (39). However, in other *in vivo* studies with fluorine 19 for MRI cell tracking, the minimal amount of tracer estimated for single-cell imaging was 0.1 pg/cell (40), which reflects the different sensitivity between both label types.

Nuclear medicine imaging techniques, such as single photon-emission computed tomography (SPECT) or PET, represent another promising modality to track stem cells *in vivo* for stroke treatment. Nuclear medicine imaging involves the use of radiotracers that can bind to different ligands. The main advantage of PET and SPECT is the extreme sensitivity, detecting molecules at the nanomolar level; however, due to the short half-life of the tracers, it is not possible to follow the cells longer than 3 or 4 days, in the best of cases (33).

Despite the high sensitivity and resolution obtained with MRI and PER probes for *in vivo* cell tracking, sometimes the image is not clear enough to visualize the graft or tracking of the targeted cells. The ambiguity of cell label detection and cell assignment that usually happens with MRI or PET can be successfully circumvented with gene reporter imaging, mainly used with bioluminescence and fluorescence reporters, a strategy commonly adopted in several studies (34, 38).

## MSCs for Stroke Treatment

Cell therapy based on the use of autologous MSCs represents one of the most promising treatments to restore function after stroke. MSCs are pluripotent stem cells that are found, in small proportion, inside the bone marrow. These cells have the ability to differentiate into different cell-type precursors (chondrocytes, osteocytes, myocytes, etc.) but their use in cell therapy is mainly based on their ability to release a wide range of bioactive molecules, with immunoregulatory and regenerative properties (41). However, one of the main causes that has limited the advance of MSCs in stroke and other neurological pathologies are the arduous protocols that are sometimes required to obtain, expand, and characterize human MSCs for clinical use (42).

Among the MSCs, due to their great regenerative potential and tissue engineering, the BMSCs are the most promising ones (41, 43). It has been shown that the i.v. administration of BMSC 24 h after stroke induces angiogenesis within the surrounding lesion area in rats. This angiogenesis is mainly due to an increase of VEGF secretion by the BMSCs, and VEGFR_2_ expression in cerebral endothelial cells (44). Also, these BMSCs are able to induce the proliferation of endogenous NSCs (45). BMSCs also have a neuroplasticity function, induced by the release of trophic factors within the affected region, enhancing restructuration processes (46). Although multiple repairing mechanisms seem to be involved in the healing effects of the BMSCs (and MSCs in general), it is well-known that their ability of releasing high amounts of extracellular vesicles is a relevant pathway by which they exert these effects (47–49).

Despite being an interesting tool for the treatment of stroke, the use of BMSCs requires bone surgical interventions that sometimes limit their use. Comparing with BMSCs, the adipose tissue-derived mesenchymal stem cells (ADMSCs) come from a more accessible source and are more abundant. Also, they have already proved their effectiveness in stroke preclinical experiments, specifically as a promising treatment for stroke-related comorbidities (15).

Although the use of ADMSCs is less studied, there are works that show their positive effects. In a report from 2013, the effect of BMSCs vs. ADMSCs was compared. Neither BMSCs nor ADMSCs have shown a reduction in infarct size or any cellular migration or engraftment, but in both cases, a reduction in cell death and an increase in the proliferation rate with an increment in the levels of VEGF, oligodendrocytes, synaptophysin, and neurofilaments at day 14, which was associated with good functional recovery, were found (50). These results show that ADMSCs present the same regenerative abilities as BMSCs, but since the source of acquisition of the ADMSCs is better, the use of these stem cells could be more suitable. Regarding the way ADMSCs exert their regenerative properties, it seems that it is mediated by the extracellular vesicles release. In a previous study, it was proved that an intravenous injection of isolated extracellular vesicles from ADMSCs produced a greater beneficial effect in rats subjected to 50 min of middle cerebral artery occlusion than the injection of just ADMSCs (51). Also, the safety of the ADMSCs has already been tested. A study in 2011 proved that an intravenous injection of ADMSCs in immunodeficient mice didn't produce any toxicity effect all along 13 weeks, even at the highest cell dose (2.5 × 10^8^ cells/kg body weight). In the same way, they tested the possibility of tumor formation along 26 weeks, but they also didn't find any evidence of tumorigenesis not even at the highest dose (2 × 10^8^ cells/kg). In a 3-month follow-up of patients with spinal cord injury to whom a single dose of hADMSCs (4 × 10^8^cells) was administered, again no evidence of any adverse effect was found (52).

The therapeutic use of MSCs has been also extended to other pathologies as hemorrhagic stroke where it has seen that administration of MSCs induce neuroprotective effects during the acute phase of the lesion (53) and functional recovery, even when cells are administered at long term (54). Moreover, MSCs have been tested in combination with recombinant tissue plasminogen activator to prevent the risk of hemorrhagic events thanks to the ability of the cells to inhibit endothelial dysfunction (55).

MSCs are also prone to cell engineering; for instance, excitatory amino acid transporter 2 (EAAT_2_) plays a pivotal role in glutamate clearance in the adult brain, thereby preventing excitotoxic effects after cerebral ischemic damage. Considering the high efficacy of EAAT_2_ for glutamate uptake, we have recently induced the expression of this transporter in MSCs for systemic administration, combining the intrinsic properties of these cells with excitotoxic protection (56).

## NSCs for Stroke Treatment

NSCs are adult stem cells present at the central nervous system that are capable of self-renewal and give birth to new neurons and glial cells, thus contributing to the plasticity and brain repair (57). These processes are enhanced within ischemic brain as a neurorepairing mechanism (58, 59). However, these processes do not occur at a rate high enough to be effective (60). Because of that, several approaches in pre-clinical studies have been performed in order to compensate or supplement these endogenous mechanisms. The most used approach consists of the administration of exogenous NSCs (61, 62). This approach has shown that i.a. administration of NSCs 24 h after stroke, in a mouse model, promotes a reduction on the inflammatory process and also reduces the BBB damage (63). This reduction of the inflammatory process was also seen in a pig stroke model, also by intraparenchymal injection, but 5 days after stroke (64). Nevertheless, the total percentage of NSCs that successfully migrate and engraft within the ischemic area is quite low. To this respect, genetic modifications that may make the cells more resistant to ischemic conditions (65, 66), such as the use of NSCs submitted to pre-conditioning conditions before administration (67, 68) or their release with various biomaterials as vehicles (69, 70), would increase this percentage. NSCs derived from human iPSCs have been also tested in rodent stroke models. The cells did not reduce stroke volume or improve behavioral recovery during the month following transplantation, but no tumor formation was observed (71). Regarding intracerebral hemorrhages, there are fewer studies, but they report improvements in functional performance after 2–8 weeks, independently of the administration route (72, 73).

## iPSCs in Stroke

On the first years after Yamanaka and Takhashi iPSC research, the experiments and preclinical studies on stroke assayed the effect of a direct injection of iPSCs into the affected region. Several studies reported improvements both in infarct volume reduction and in functional recovery (74–76). Also, improvements in the neurological function and survival rate in hemorrhagic stroke were reported (77). Most of these improvements attributed to the iPSCs were due to a differentiation of these iPSCs into different adult stem cells in the affected region (78, 79).

Although some studies have reported that iPSC-derived NSCs transplanted in the brain of mice subjected to stroke have no tumorigenicity risk (29) or iPSCs in intracerebral hemorrhage stroke (77), one of the main limitations of iPSCs in order to achieve a future translationality to the clinical is the formation of teratomas or tumorigenicity in the following weeks after cell administration (80). This is due to the environmental effects of the niche where the iPSCs are implanted. Formation of teratomas can also occur by transformation of residual iPSCs that remain on the implanted area and can become benign teratomas after some time (81, 82). To this respect, there are some studies that tried to solve this issue. For example, a study published by Chen et al. compared the formation of teratomas after the injection of iPSCs with and without fibrin glue as vehicular agent into the subdural region instead of its injection right into the cortex. In the injection of iPSCs by themselves, there was always formation of teratomas after 4 weeks, whereas in the ones injected with fibrin glue, there was not. The authors pointed out that this was not only due to the fibrin glue but also because the subdural region was not a niche appropriate enough to induce an uncontrolled growth of the iPSCs (74). Besides, another study showed the possibility of inhibiting the formation of tumors due to this residual iPSCs by treatments with inhibitors of specific pro-apoptotic routes of stem cells, inducing their apoptosis and erasing them; meanwhile, the derived differentiated cells survived and maintained their functionality (82).

In conclusion, although the therapy with iPSCs is yet on preclinical experimentation, the use and the evaluation of this cell therapy is increasing significantly every year, since this new approach is a faster way to generate human stem cells (83–85). However, because this is a relatively recent discovery, the use of iPSCs for cell therapy still presents some issues that need to be explored such as the optimal time window, optimal cell/dose, or tumorigenicity (86). In case of stroke, it is also not clear if the therapeutic effect observed in ischemic animal models treated with iPSCs is mediated by cell renewal and/or by replacement of the damaged tissue (87) as the transplanted cells disappear few weeks after administration (30).

Currently, the proper differentiation iPSCs to the cell line of interest (e.g., neuronal, epithelial, etc.) with reduced division capacity seems to be the most convenient way to address these limitations.

## iPSCs and CADASIL

Within the field of stroke, 25–30% of events are caused by cerebral small vessel diseases (SVDs). Although stroke is a well-studied disease and its mechanisms and underlying processes are well-known, there are some pathologies that can be the cause of cerebral infarcts like SVDs, which present a lack of approaches and understanding (88). Despite the impact that these SVDs have on the brain, nowadays there are no specific treatments for them. Furthermore, there are limited therapeutic options for secondary prevention compared with those for other common causes of stroke. As a result, even though it is the cause of the onset of the stroke in many cases, currently there are no solutions or treatments available. The few studies that have been carried out on these diseases point to the study of monogenic variants of SVDs, in order to provide valuable insights into the molecular mechanisms underpinning idiopathic SVDs.

One of these variants is the cerebral autosomal dominant arteriopathy with subcortical infarcts and leukoencephalopathy, commonly known as CADASIL. CADASIL patients develop leukoencephalopathy, migraines with aura, recurrent ischemic strokes, motor disability, and dementia as main symptoms. Currently, there is no treatment for this disease. These symptoms are caused by progressive weakness of the small brain vessels, which coincide spatially with granular osmiophilic material (GOM). This vessel weakness is due to a continuous aberrant accumulation of the extracellular component of the Notch3 protein membrane in the GOM (89). In CADASIL, the *NOTCH3* gene presents a mutation in one of its exons that results in a loss or a gain of a cysteine residue. This mutation leads to an incorrect process of the metabolic pathway when the Notch3 signal is triggered, which ends with the accumulation of the extracellular domain of the protein in the GOM (89, 90).

Although there are several mouse models of CADASIL (knockouts for several and different mutations), it has been impossible to find any solution to the progressive accumulation of the extracellular domain of Notch3. In this line, iPSCs may shed a light over this disease. The main cell types affected in CADASIL are the vascular smooth muscle cells (VSMCs), which express the mutation, and the vascular endothelial cells (VECs), which interact with the VSMCs. With cell modeling, it would be possible to generate these cells from reprogrammed iPSCs from adult somatic cells of a CADASIL patient and perform mono and co-cultures that would allow one to study and better understand the molecular mechanisms of this disease and clarify the confusing and sometimes contradictory information that the mouse models provide (90). Also, over these cultures, a drug screening may be performed to see if any drug is able to slow down or even stop the accumulation of the extracellular domain of Notch3. Lastly, it would also be possible, by genetic editing, to repair the mutation within the iPSCs and, once differentiated into VSMCs, administer them in *Notch3* knockouts mice to see if the healthy VSMCs replace the damaged endogenous ones.

At the moment, there are just two recent studies where, for the first time, the reprogramming of adult somatic cells from a patient with CADASIL to iPSCs was made, proving that *NOTCH3* mutation is not a limitation to the reprogramming (11, 12). The generation of this iPSC line offers an unprecedented opportunity for studying and modeling both CADASIL and other pathologies related to the vascular risk of stroke.

## Clinical Trials

Currently, there are no clinical trials for stroke with iPSCs. This is due to the fact that the iPSCs are yet in a relatively early stage of study, and that they present several problems yet to solve and previously described regarding their safety.

However, in the last decade, different clinical trials have been carried out or are being carried out all over the world, mainly with MSCs (Table 1). These trials are shedding light about quantities, routes of administration, and efficacy at different times with stem cells, as well as their safety.

**Table 1:** Main clinical trials that are currently being carried out or that have already finished.

**Name of trial**	**Design**	***N* = Patients recruited *n* = patients treated**	**Cells**	**Time**	**Dosage (cells)**	**Deliver**	**Follow-up**	**Efficacy**	**Adverse effects**
Intra-arterial stem cells in subacute ischemic stroke (NCT00761982) (91)	RCT	Subacute MCA stroke *N* = 20 (*n* = 10)	BM-MNCs	5–9 days	1.59 × 10^8^ cells at 0.5 to 1 mL/min	I.A.	6 months	Inconclusive	N/A
Stem cell therapy for acute ischemic stroke patients (inVeST) (NCT0150177) (92)	RCT	Subacute stroke *N* = 120 (*n* = 58)	BMSCs	18.5 days	2.8 × 10^8^ cells	I.V.	6 months	No	Safe
Reparative therapy for acute ischemic stroke with allogeneic mesenchymal stem cells from adipose tissue: a safety assessment (NCT01678534) (93)	RCT	Acute stroke *N* = 20 (*n* = 10)	MSCs	≤2 weeks	1 × 10^6^ cells/kg at 4–6 mL/min	I.V.	2 years	↑Neurological outcomes	Safe
Safety/feasibility of autologous mononuclear bone marrow cells in stroke patients (NCT00859014) (94)	Open-label	Acute MCA stroke *N* = 10	BM-MNCs	24–72 h	7 × 10^6^ to 1 × 10^7^ cells/kg over 30 min	I.V.	6 months	Inconclusive	Safe
Intravenous transplantation of mesenchymal stem cells preconditioned with early phase stroke serum: current evidence and study protocol for a randomized trial STARTING-2 (NCT01716481) (95)	PROBE	Acute and chronic stroke *N* = 60 (*n* = 40)	MSCs	≤90 days	1 × 10^6^ cell/kg	I.V.	3 months	Going on	N/A
Safety and efficacy of multipotent adult progenitor cells in acute ischemic stroke (MASTERS): a randomized, double-blind, placebo-controlled, phase 2 trial (NCT01436487) (96)	RCT	Acute stroke *N* = 129 (*n* = 67)	MAPC	24–48 h	4 × 10^8^ or 12 × 10^8^ cells	I.V.	3 months	No	Safe
Intra-arterial immunoselected CD34+ stem cells for acute ischemic stroke (NCT00535197) (97)	Prospective, open-label	Severe anterior circulation stroke *N* = 5	Autologous immunoselected CD34+ stem/progenitor cells	≤7 days	1 × 10^8^ cells over 10 min	I.A.	6 months	↑Clinical outcomes	Safe
Human neural stem cells in patients with chronic ischemic stroke (PISCES): a phase 1, first-in-man study (NCT01151124) (98)	Open-label	Stable disability after stroke *N* = 13 (*n* = 11)	CTX0E03	6–60 months	2 × 10^8^, 5 × 10^8^, 10 × 10^8^, 20 × 10^8^ cells at 5 μL/min	Putamen	≥24 months	↑NIHSS	Hyperintensity in brain
Clinical outcomes of transplanted modified bone-marrow-derived mesenchymal stem cells in stroke: a phase 1/2a trial (NCT01287936) (99)	Open-label	Stable, chronic stroke *N* = 18	BMSCs	6–60 months	2.5 × 10^8^, 5 × 10^8^, 10 × 10^8^ cells. Deposits of 20 μL of cells over 10 min each	Peri-infarct zone	24 months	↑Clinical outcomes endpoints	Safe

Some of the already finished trials, despite their limitations, show results that seem to point into the right direction. For example, the InVeST trial designed to evaluate the effect of intravenous BMSCs injection did not show a beneficial effect but proved the safeness of BMSCs use in humans (92). In 2016, another clinical trial with BMSCs, apart from corroborating the safeness of BMSCs administration, found improvements in clinical outcome (NIHSS, ESS, and Fugl–Meyer scores) with stroke patients. However, it was a non-randomized small trial (*n* = 18), so its results should be taken with caution (99). On the other hand, the PISCES study (98) tried to prove the beneficial effect of human NSCs in patients who have had a first ischemic stroke in the last 6–60 months. After a follow-up of 2 years, they found improvements in neurological function and no adverse effects in cell dosage up to 20 million cells. Nonetheless, it was also a small trial (*n* = 13, split into four different groups depending on cell dosage), and in two patients, a slight increase in hyperintensity in pre-existing peri-infarct white matter hyperintensity was found, although there were no clinical changes associated to this MRI changes.

As it can be deduced from the trials above, in general, the ongoing and finished clinical trials present in their majority some limitations that must be solved in the subsequent trials. While all trials provide the number of transplanted cells and their route of administration, some of them omit the cell dosage. On the other hand, some of the clinical trials in which no benefit is found point to a late injection time. To this regard, choosing the first days after stroke as the moment for injection may be a critical point in order to obtain benefits from the injection, but it also involves the risk that the action of the cytokines that happens during the first days after stroke may mask the real effect of the cell dosage. Also, there is the risk that the patient could be subjected to unforeseen surgeries, like hemicraniectomy, that may alter the cell dosage effect. Besides, and in the same way as happens in preclinical experiments, the choice of the administration route is also a key factor, and while new trials base their choice in previous trials, there is still work to do to establish secure criteria for route of administration and cell dosage based on the cell type.

Finally, current clinical trials are, in general, small trials waiting for their expansion or the creation of new and bigger ones with a higher recruitment capacity that may corroborate the results obtained by these pioneering trials.

## Discussion

In recent years, a multitude of studies about stem cells and iPSCs are being carried out within the field of cerebrovascular diseases. These studies have been proving the efficiency to induce both neuroreparative and neuroprotective effect, but they also have permitted to refine some important issues like the number of cells and the most appropriated route of administration depending on the cell type, which has allowed various centers all over the world to start different clinical trials, getting closer to the final goal, a consolidated cell therapy for stroke. However, the ongoing trials are yet too small to be really conclusive, and bigger ones with a wider number of patients would have to be carried out in order to achieve more conclusive results.

There are still several important limitations to consider in case cell therapy on stroke becomes a reality in clinical practice. Stroke is a cerebrovascular pathology and, as such, it is not a chronic disease that may be treated over time. In stroke, the therapeutic window for recanalization or neuroprotective treatments comprehends the first hours after stroke, and that is why the treatment should be ready at the arrival of the patient to the hospital. In this case, the availability of stem cells would not reach this period of time, since their use would implicate their previous extraction from the patient for a neuroreparative treatment of these cells; it would also be hardly achievable to have the cells on time, since the reprogramming and/or differentiation can take up to 6 months easily. To solve this problem, researchers addressed the possibility of establishing universal stem cell banks, derived from an universal pull of iPSCs coming from a population set that would cover all the combinations of the human leucocyte antigen alleles (HLA), avoiding any chance of immune rejection (100, 101).

Another problem to take into consideration would be the future commercialization of the cells. The pharmaceutical industry tends to be interested in drugs of easy distribution and maintenance, while the cells, being living organisms, would require a special treatment on this regard. This would not make them especially attractive for the industry, and even if the maintenance cost could be handled by the hospitals, the distribution cost would still exist.

In the last few years, a new field of research has been developed, focused on the extracellular vesicles released by adult stem cells, since it is through them that they exert their main benefits. The use of these vesicles will replace the need to use adult stem cells, obviating their associated problems. However, this technology comes with other problems that need to be solved, like the vesicle dosage (which is unsettled), time and mode of application, or their biodistribution (102).

## Future Perspectives and Alternatives

Cell therapy (especially MSCs and NSCs) in stroke based on the use of stem cells has advanced considerably but, to become a reality, while wider and deeper clinical trials are carried out, limitations such as immediate availability and distribution of stem cells for clinical use must be overcome. Extracellular vesicles released by MSC are emerging as a novel alternative to cell therapy as they could have similar beneficial effects to MSC but with lower risk effect (in terms of vessel occlusion); they are easier to handle with respect to cells and can also be used as biomarkers to evaluate stroke recovery after cell therapy.

The potential risk of tumorigenicity associated with the direct use of iPSCs as cell therapy for human treatment represents the main limitation for immediate clinical use. The improvement in genome editing techniques, such as CRISPR/Cas9, has expanded the use of iPSCs derived from patients to generate healthy cells that can provide a useful channel for precision therapy with potentially lower tumorigenic risk. The use of the iPSC-based disease model in stroke-related pathologies as CADASIL is expected to provide potential therapeutic strategies for cerebral SVDs.

## Author Contributions

Drafting of the article was done by HF-S, AB-C, and FC. Manuscript revision was done by JC and FC.

### Conflict of Interest Statement

The authors declare that the research was conducted in the absence of any commercial or financial relationships that could be construed as a potential conflict of interest.
